# Acupuncture for Subthreshold Depression: Study Protocol for a Randomized Controlled Trial

**DOI:** 10.3389/fpsyt.2021.772360

**Published:** 2022-01-27

**Authors:** Jin Xian, Ling Wang, Mi Sun, Xue Wang, Xiao-Ming Zang, Hui-Juan Yu, Xin Zhang, Bin Cheng, Qi-Wen Tan

**Affiliations:** ^1^Affiliated Hospital of Shandong University of Traditional Chinese Medicine, Jinan, China; ^2^Shandong University of Traditional Chinese Medicine, Jinan, China

**Keywords:** subthreshold depression, acupuncture, protocol, randomized controlled trial, sham acupuncture

## Abstract

**Background:**

Subthreshold depression refers to a state of mental sub-health that has depressive symptoms but does not meet the diagnostic criteria for major depressive disorder. It is a strong risk factor for depression and is related to an increase in suicide and mortality. Studies suggest that acupuncture may be effective in the treatment of subthreshold depression, but no evidence has proven that the efficacy of acupuncture for subthreshold depression is a specific therapeutic effect or a placebo effect.

**Methods:**

This will be a prospective, randomized, controlled, and double-blind study of 64 patients with subthreshold depression. They will be randomly assigned as verum or minimal acupuncture group members. Patients in the verum acupuncture group will receive acupuncture at the acupoints, and those in the minimal acupuncture group will receive minimal acupuncture at non-acupoints. The interventions will be delivered over an 8-week period. The primary outcome measure will be the Hamilton Depression Scale-17 (HAMD-17). The secondary outcome measures will be the 9-item Patient Health Questionnaire (PHQ-9), 7-item Generalized Anxiety Disorder (GAD-7), and SF-12v2 Health Survey. The assessments will occur at baseline, 4 weeks, 8 weeks, and during a follow-up period.

**Discussion:**

The protocol uses a randomized controlled trial to examine the effectiveness of acupuncture for subthreshold depression and to further study the mechanisms of its effect.

## Background

Subthreshold depression refers to a state of mental sub-health that has depressive symptoms but does not meet the diagnostic criteria for major depressive disorder (MDD) in the Diagnostic and Statistical Manual of Mental Disorders (DSM) (1). According to the results of the American epidemiological survey (2), 20% of the general population has depressive symptoms that belong to subthreshold depression. Braam (3) surveyed 14,200 elderly people in 7 European cities and found that the prevalence of subthreshold depression is at least 2–3 times higher than that of depression, of which ~8% develop depression each year. In China, the incidence of subthreshold depression among college students has reached 36.56% (4). A survey by the World Health Organization showed that depression is the second leading cause of disability (5), and it will become the prime global disease burden in 2030 (6). Subthreshold depression is a strong risk factor for depression (7), which may occur alternately with MDD over time (8). Although the symptoms are mild, they are still related to an increase in suicide and mortality (9). In addition, the degree of depression is positively correlated with cardiovascular and cerebrovascular diseases, such as coronary heart disease and stroke (10). Therefore, timely detection, diagnosis, and treatment of subthreshold depression will delay or reverse the development of MDD to effectively reduce the harm of depressive symptoms. A systematic review showed that antidepressants had no significant advantage over placebo in patients with minor depression (11), and similar conclusions were drawn in randomized trials (12). Therefore, limited evidence suggests that antidepressants may be beneficial to patients with more severe depression (13) but are often accompanied by severe adverse reactions.

Acupuncture, as a non-drug therapy, has been widely used in the treatment of depression (14–16). A small sample study showed that electroacupuncture (EA) and cognitive behavioral therapy (CBT) could ameliorate the Hamilton Depression Scale-17 (HAMD-17), Center for Epidemiologic Depression scale (CES-D), and WHO Quality of Life-Brief version scores of subthreshold depression, but no significant differences were found between the EA and CBT groups (17). Another study showed that acupuncture can reduce HAMD-17 and CES-D scores compared with the blank control (18). These studies suggest that acupuncture may be effective in the treatment of subthreshold depression. Evidence has shown that placebo drugs or CBT can reduce depression symptoms, although it remains unclear whether the effect of acupuncture on subthreshold depression is a specific therapeutic effect or a placebo effect. Since no evidence has emerged to address this question, we designed this study.

## Methods

This protocol was designed according to the standard protocol items: recommendations for interventional trials (SPIRIT) checklist and Standards for Reporting Interventions in Clinical Trials of Acupuncture (STRICTA) 2010 checklist of information to include when reporting interventions in a clinical trial of acupuncture. The study protocol (version: 20210722-2.0) was approved by the Institutional Review Board (IRB) of the Affiliated Hospital of Shandong University of Traditional Chinese Medicine (2021-055-KY), and this study was registered in the Chinese Clinical Trial Registry (ChiCTR2100049660). If there are any changes in the study design, the Ethics Committee will be immediately informed.

### Trial Objective

Current studies have shown that acupuncture can relieve the clinical symptoms of subthreshold depression, but it is not clear whether the effect of acupuncture on subthreshold depression is caused by the placebo effect. Therefore, the objective of this study is to evaluate the efficacy of acupuncture in the treatment of subthreshold depression by comparing verum acupuncture and minimal acupuncture and to verify whether there is a placebo effect. In addition, we will explore the mechanism of acupuncture in the treatment of subthreshold depression.

### Trial Design

**Figure 1:**
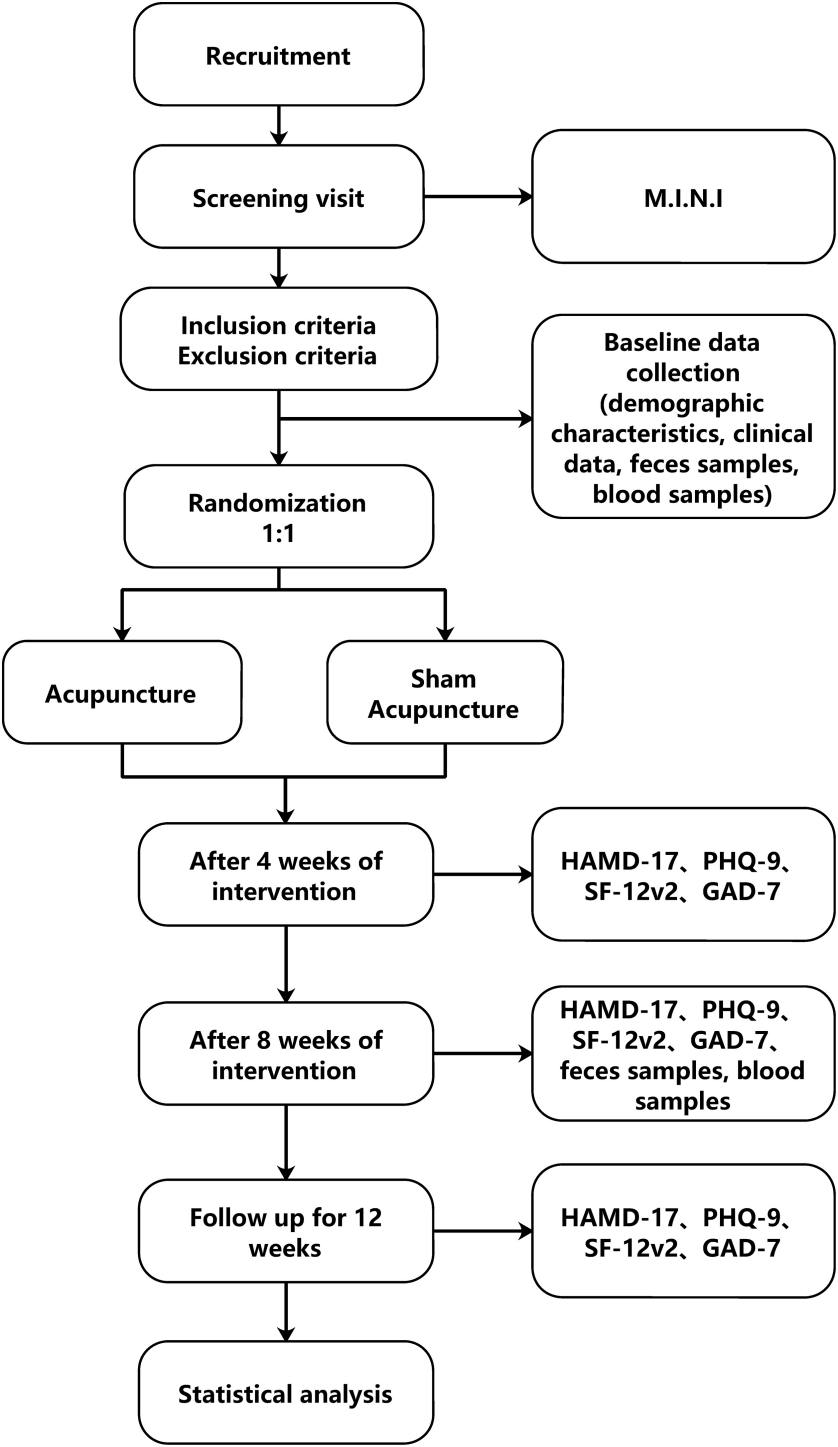
Flow chart of study design. M.I.N.I, Mini International Neuropsychiatric Interview; HAMD-17, Hamilton Depression Scale-17; PHQ9, The 9-item Patient Health Questionnaire; GAD-7, 7-item Generalized Anxiety Disorder; SF12v2, SF-12v2 Health Survey.

This study will be a single center, randomized, double-blind clinical trial with two treatment groups (verum acupuncture and minimal acupuncture). The flow chart in Figure 1 shows more details regarding the clinical procedures.

### Setting

The trial will be conducted at the preventive and health care center of the Affiliated Hospital of Shandong University of Traditional Chinese Medicine (Jinan, China).

### Participants

Participants will be recruited by advertisement. Interested volunteers can contact the research assistants by WeChat or phone. All volunteers will be screened by applicants according to the inclusion and exclusion criteria. The research assistants will introduce the research process, consent forms to the volunteers in detail, and the designated plan of each group. Eligible volunteers were invited to participate in the study, signed an informed consent form, and then arranged for a baseline assessment.

### Inclusion Criteria

Participants will be included if they meet the following criteria:

1. The Mini International Neuropsychiatric Interview (MINI version 5.0) will be used to identify subthreshold depression patients who have depressive symptoms but do not meet the major depression disorder criteria according to the Diagnostic and Statistical Manual of Mental Disorders (Fifth Edition) (DSM-V);
2. Aged between 18 and 60 years;
3. The score of HAMD-17:7 ≤ HAMD <17;
4. No treatment related to depression has been received in the past 2 weeks;
5. Antibiotics, probiotics, yogurt, and other fermented dairy products were not taken before random sampling;
6. Voluntarily participating in this trial with a written informed consent form.

### Exclusion Criteria

Participants with the following conditions will be excluded:

1. Meeting the criteria for major depressive disorder, bipolar disorder, or psychotic disorder according to DSM-V;
2. Patients with severe liver and kidney damage, severe arrhythmia, or cardiac insufficiency;
3. Pregnant women;
4. Depression caused by psychoactive, non-addictive substances, or brain diseases;
5. Patients have suicidal attempt or behavior;
6. Patients who cannot tolerate acupuncture;
7. Patients who are difficult to communicate with or do not cooperate with the researcher;
8. Other situations determined by the researchers to be unsuitable for observation.

### Elimination and Withdrawal Criteria

1. Misdiagnosis;
2. Self-use of other related drugs, antibiotics, probiotics, yogurt, and other fermented dairy products;
3. Subjects dropped out of the study, had difficulty cooperating with the treatment, or were lost to follow-up in the later stage.

### Observation Criteria That Would Stop the Trial

1. Cases with serious complications or other serious diseases during the study and if urgent measures are needed.
2. The progression of disease during treatment.

### Sample Size Estimation

This pilot study aims to assess the efficacy of acupuncture for subthreshold depression as well as the feasibility of a further large clinical trial. The HAMD-17 was used as the main effect indicator to calculate the required sample size. According to relevant published literature (19, 20), the difference in HAMD-17 scores between the acupuncture and placebo groups was 4.87, and the standard deviation was 1.99 in the acupuncture group and 7.18 in the placebo group. Group sample sizes of 27 and 27 achieve 90.75% power to reject the null hypothesis of equal means and with a significance level (alpha) of 0.050 using a two-sided two-sample unequal-variance *t* test by PASS 15 software. Assuming a dropout rate of 15%, 64 participants are necessary, with 32 participants in each group.

### Randomization and Allocation Concealment

Independent statisticians will use the block randomization method with R 4.0.5 software and the blockrand R package. An independent clinical trial researcher will implement the allocation schedule using opaque sealed envelopes. A random number will be assigned after the participants meet all inclusion criteria and complete the baseline assessment. The Clinical Research Coordinator (CRC) will be responsible for enrolling participants, obtaining informed consent, and requesting randomization.

### Blinding

Blinding of acupuncturists is not possible due to the therapeutic characteristics of acupuncture; thus, the acupuncturist who provides treatment for patients is unable to be blind to treatment allocation. Participants and all other researchers will be blind to the treatment allocation plans, including data analysts and outcome assessors. To ensure the implementation of the blind method, patients will avoid communicating with each other during the trial period, and an isolatable treatment bed will be used for acupuncture treatment to avoid patients seeing treatment of others. Treatment groups will be defined as Group A and Group B during the statistical analysis of the data.

### Intervention

Verum acupuncture and minimal acupuncture will be performed by certified acupuncturists who have worked at least 5 years in clinics. Before the trial, all acupuncturists will be required to receive special training to fully understand the performance of the treatment and will receive a pamphlet detailing the standardized operation. The training contents include acupoint and non-acupoint positioning methods, acupuncture manipulation and minimal acupuncture manipulation. A study investigator will communicate with the participants every week in advance of trial commencement via phone and WeChat to remind them of their visit. Other antidepressant treatments will be prohibited during the trial.

#### Verum Acupuncture

The treatment plan for acupuncture is dependent on the Acupuncture Therapeutics textbook (21) and the clinical experience of acupuncture experts. Subjects randomized to the verum acupuncture group will receive 24 sessions of acupuncture treatment (3 times a week for 8 weeks; 30 min every session). Baihui (GV20), Yintang (GV29), Shenmen (HT7), Taichong (LR3), Tianshu (ST25), and Zusanli (ST36) will be used in the verum acupuncture group. Acupuncturists will use sterile needles (Huatuo disposable acupuncture needle) 0.30 × 40 mm in size in the acupoints, twirling and lifting will apply for 30 s at each acupoint to achieve the typical acupuncture sensation of Deqi (soreness, numbness, distension, and heaviness), and needles will be retained in place for 30 min.

#### Minimal Acupuncture

Non-acupoints (Table 1) without antidepressant therapy will be used to provide acupuncture for subjects in the minimal acupuncture group. Acupuncturists will use sterile needles 0.30 × 25 mm in size punctured 2 mm in depth without achieving a Deqi sensation (feel nothing after the needle is punctured). The course of treatment will be the same as that of the verum acupuncture group. After the follow-up period, 24 sessions of acupuncture treatments will be compensated for free according to the subject's wishes.

**Table 1:** Location of non-acupoints in minimal acupuncture group.

**Non-acupoints**	**Location**
NA1	In the outside of the forearm, 1 cm outside the midpoint of the line between Yangchi (TE4) and Waiguan (SI4)
NA2	In the outside of the forearm, 1 cm outside the midpoint of the line between Shousanli (LI10) and Quchi (LI11).
NA3	In the abdomen, 1 cm outside the midpoint of the line between Daheng (SP15) and Fushe (SP13)
NA4	In the outside of the calf, 1 cm below the midpoint of the line between Zusanli (ST36) and Yanglingquan (GB34)
NA5	In the back of the foot, the midpoint of the line between Qiuxu (GB40) and Jiexi (ST41)

*NA, non-acupoints*.

### Outcomes

**Figure 2:**
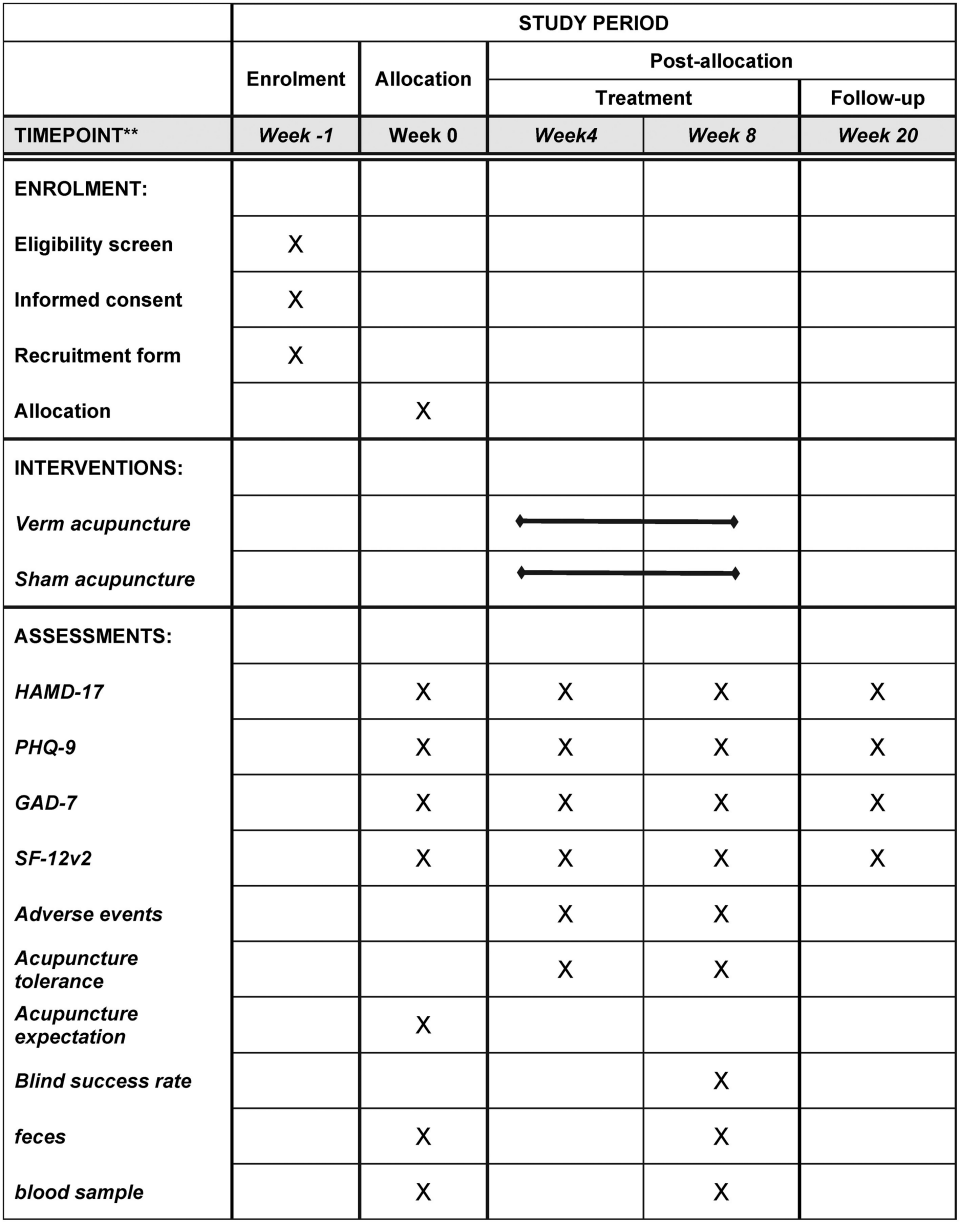
SPIRIT figure.

Figure 2 summarizes the outcomes and time points of data collection during the trial.

#### Primary Outcome Measurement

The primary outcome will measure changes in the HAMD-17 scores between baseline and 8 weeks after randomization. The Hamilton Depression scale (22), developed by Professor Max Hamilton in 1960, is a widely used depression scale. It includes cognitive impairment, block, despair, anxiety/somatization, sleep disorder, weight, and diurnal changes. It is mainly used to evaluate the severity of depressive symptoms in adult patients with depression and the curative effect before and after clinical trials. The higher the score, the more serious the degree of depression.

#### Secondary Outcomes

##### HAMD-17 at Other Timepoints

The primary outcome measurement is the HAMD-17 at week 8 after randomization; however, it will also be used at weeks 4 and 20 after randomization.

#### Symptom Assessment

Compared with the baseline period, the changes in PHQ-9, SF-12v2, and GAD-7 scores and the proportion of people who developed moderate depression (HAMD-17>17 or PHQ-9>10) at weeks 4, 8, and 20 will be measured. The PHQ-9 is a self-reporting tool to evaluate 9 depressive symptoms according to the DSM-IV depression criteria. It has a total of 9 items, each with a score of 4 (0–3), leading to a total score of 0–27. This score can be used to describe the patient's symptoms (23). The Chinese version of the PHQ-9 has also proven its reliability and validity (24, 25). The SF-12v2 is a scale widely used to measure quality of life (26). At present, the second edition is widely used and mainly evaluates the two general dimensions of physical health (PCS) and mental health (MCS). The higher the score, the better the health-related quality of life. The GAD-7 is a self-reporting tool that assesses anxiety symptoms and aims to measure the frequency of anxiety symptoms over the past two weeks (27). Scores of 5, 10, and 15 points represent thresholds for mild, moderate, and severe anxiety symptoms, respectively.

#### Evaluation of Acupuncture Tolerance

**A** visual analog scale (VAS) will be used to evaluate the discomfort of acupuncture after the first and last treatment. We will use a three-point method to evaluate acupuncture receptivity, which is unacceptable (0 points), acceptable (1 point), or easy to accept (2 points). Those who cannot accept acupuncture will write down the reasons clearly.

#### Evaluation of Acupuncture Expectation

Before the first treatment, we will use the acupuncture expectancy scale to evaluate the expectation of acupuncture in the subjects. The higher the score, the higher the expectation for the efficacy of acupuncture.

#### Blind Success Rate Evaluation

The percentage of patients who choose acupuncture will be compared between the two groups at the end of the last treatment.

#### Adverse Events

The contents include broken needles, missing needles, dizziness after acupuncture, unbearable acupuncture pain (VAS ≥ 8), severe post-acupuncture pain lasting more than 2 h (VAS ≥ 4), local hematoma, infection, or abscess. Other unforeseen adverse events will be recorded at all trial periods.

#### Exploratory Outcomes

Patient blood samples and feces will be collected at week 0 and week 8 after randomization and will be used to establish a biological sample bank, which may help to promote the research program and explore the therapeutic mechanism of acupuncture intervention in subthreshold depression.

Normally distributed continuous variables are presented using the means and standard deviation.

### Data Collection

All participant data will be recorded on the original case report forms (CRFs). To ensure the accuracy of the data, the data will be entered into Excel 365 by two independent data entry clerks. All paper data related to the trial will be saved, and electronic documents will be stored in a password protected computer. All research documents, including paper and electronic documents, will be preserved for at least 5 years after publication. If readers have any questions about the data we publish, they can contact the first author or corresponding author for the original data. Private information, such as the patient's name, age, and phone number, will be protected and will never be disclosed to anyone. Feces and blood samples will be collected in strict accordance with standardized procedures and stored in a −80°C freezer to establish a biological sample bank.

### Statistical Analyses

R 4.0.5 software will be used for statistical analysis by an independent statistician who will not participate in the study. Two-tailed *p*-values < 0.05 will be considered statistically significant. Normally distributed continuous variables will be presented using the means and standard deviation, and non-normal variables will be presented as the medians and interquartile range. All efficacy analyses will be performed using the intent-to-treat (ITT). Missing data will be replaced according to the principle of the last observation carried forward method (28). The per-protocol (PP) population will be analyzed to determine the consistency of the results. The data before and after treatment in each group will be compared based on a paired *t* test or Wilcoxon signed-rank test, and the comparison between groups will be performed using an independent sample *t* test or Mann–Whitney *U* test. Categorical variables will be analyzed using the χ^2^ test or Fisher's exact test. For comparisons of the primary outcomes across the groups, we will use analysis of covariance with the baseline HAMD-17 to adjust. A generalized linear mixed model will be used for repeated measures, setting group and time as fixed factors and patient as a random factor; the interaction effects of group^*^time will be included in the model.

### Quality Control

To ensure the smooth progress of the research, before the formal start of the clinical trial, we will hold special clinical training to unify the clinical researchers. We will focus on the implementation plan of the project and the standard operating procedures so that each clinical researcher is familiar with the research process and specific implementation details, to improve the consistency of internal observation and the consistency between observers and ensure the reliability of clinical research conclusions. To reduce the withdrawal of patients, we will improve and maintain patients' good compliance and urge patients to stick to the treatment as much as possible during the study. All dropout subjects will be asked to state their reason, and these reasons will be analyzed.

Only researchers participating in clinical trials may have access to the subjects' personal medical records, and they will sign the investigator's statement or confidentiality commitment to include confidential content. The ethics committee has the right to inspect clinical trial records. Data will be processed in an anonymized way, and information that can identify the subject's individual identity will be omitted.

## Discussion

There is a lack of clear diagnostic criteria for subthreshold depression in the ICD-10 and the DSM-V. Thus, subthreshold depression is mainly diagnosed with identification tools for the diagnosis of depression, such as the MINI-International Neuropsychiatric Interview and HAMD-17. As a special critical state of disease, subthreshold depression has a significantly higher risk of transforming into depressive disorder than the general population (29). Compared with healthy people, people with a low level of education, low social support, history of serious chronic diseases, poor health, greater stress in the past 6 months, and living in poverty were more likely to suffer from subthreshold depression (30). Early intervention can relieve symptoms, reduce the risk of severe depression, and prevent progression to other adverse consequences. However, the current evidence does not support the use of drugs to intervene in patients with subthreshold depression (11, 12). Psychotherapy, such as cognitive behavioral therapy (CBT), is the main method to intervene in subthreshold depression at present (31, 32). The economic cost of patients receiving CBT intervention is still high, and the standardization and consistency of CBT intervention vary greatly among different centers (33). Acupuncture, as a safe non-drug therapy, has the advantages of simple operation and low treatment cost. It has been widely used to treat different psychiatric conditions, including depressive disorder (14, 15, 34). To date, few studies have investigated acupuncture for subthreshold depression (18, 35). The main principles for the treatment of depression in the theory of traditional Chinese medicine are tranquilize the mind and disperse stagnated hepatoqi. GV20, GV29, and HT7 will be used to tranquilize the mind, and LR3, ST25, and ST36 to disperse stagnated hepatoqi.

The goal of this RCT is to assess the effectiveness of acupuncture compared with minimal acupuncture to prove whether there is a placebo effect for subthreshold depression. In addition, the gut-brain axis plays an important role in depression (36, 37). However, there is no evidence that acupuncture interferes with subthreshold depression through the gut-brain axis; therefore, we will collect blood and stool samples from the subjects to establish a biological sample bank. If our clinical trials prove that acupuncture in the treatment of subthreshold depression is not just a placebo, we will use this biological sample bank to explore the mechanism of acupuncture for subthreshold depression.

The primary limitation of our trial is that acupuncturists cannot be blinded. Second, as a preliminary exploratory study, this study was designed as a single center study. If the study is effective, we will conduct further multicenter research.

## Trial Status

The protocol version is 2.0 (in July 22, 2021.) The trails have begun on July 26, 2021 and approximately completed on July 26, 2022.

## Ethics Statement

All participants must grant written informed consent once trial details are fully presented. The authors are accountable for all aspects of work, ensuring that questions related to accuracy or integrity of any part are appropriately investigated and resolved. The ethical approval (2021-055-KY) was given by the Institutional Review Board (IRB) of Affiliated Hospital of Shandong University of Traditional Chinese Medicine.

## Author Contributions

JX, LW, and XZ: conception and design. Q-WT: administrative support. H-JY, LW, and X-MZ: provision of study materials or patients. MS and XW: collection and assembly of data. JX and BC: data analysis and interpretation. All authors drafting of manuscript and final manuscript approval.

## Conflict of Interest

The authors declare that the research was conducted in the absence of any commercial or financial relationships that could be construed as a potential conflict of interest.

## Publisher's Note

All claims expressed in this article are solely those of the authors and do not necessarily represent those of their affiliated organizations, or those of the publisher, the editors and the reviewers. Any product that may be evaluated in this article, or claim that may be made by its manufacturer, is not guaranteed or endorsed by the publisher.

## Abbreviations

MDD: major depressive disorder
DSM: Diagnostic and Statistical Manual of Mental Disorders
EA: electroacupuncture
CBT: cognitive behavioral therapy
HAMD-17: Hamilton Depression Scale-17
CES-D: Center for Epidemiologic Depression scale
PHQ-9: The 9-item Patient Health Questionnaire
GAD-7: 7-item Generalized Anxiety Disorder
SF-12v2: SF-12v2 Health Survey
VAS: visual analog scale.
